# Albumin Protein Cleavage Affects the Wear and Friction of Ultra-High Molecular Weight Polyethylene

**DOI:** 10.3390/lubricants5030033

**Published:** 2017-08-17

**Authors:** Yasha Dwivedi, Michel P. Laurent, Shravan Sarvepalli, Thomas M. Schmid, Markus A. Wimmer

**Affiliations:** Department of Orthopedic Surgery, Rush University Medical Center, Chicago, IL 60612, USA

**Keywords:** albumin, cleaved albumin, polyethylene, UHMWPE, CoCrMo alloy, knee prostheses, Miller-Galante II, wear, pin-on-flat, knee simulator, particles, debris

## Abstract

It is well established that the total protein concentration and albumin-to-globulin ratio influence the wear of ultra-high molecular polyethylene (UHMWPE, “polyethylene”) in joint prostheses. A factor on wear not yet studied, but of possible clinical relevance, is protein cleavage. Such cleavage is expected in the presence of an inflammatory response and as a result of wear processes at the articular interface. The aim of this study was to compare the tribological behavior of polyethylene articulated against an orthopedic wrought CoCrMo alloy for three lubricants: cleaved albumin, uncleaved albumin, and newborn calf serum (control). We hypothesized that the cleavage of albumin will increase the friction and wear rate of polyethylene, with a concomitant roughening of the polymer surface and the generation of larger wear debris particles. Cleavage of the bovine albumin into five fragments was performed by digestion with cyanogen bromide. In pin-on-flat (POF) wear tests of polyethylene pins made of Ticona GUR^®^ 1020/1050 against CoCrMo alloy discs, the cleaved albumin led to the lowest polyethylene wear and highest friction coefficients, whereas albumin led to the highest wear rates. In knee simulator tests, the albumin lubricant also led to a 2.7-fold increase in the tibial insert wear rate compared to the regular bovine serum lubricant (a wear rate for the cleaved albumin could not be obtained). The generated polyethylene wear particles were of increasing size and fibrillar shape in going from serum to albumin to cleaved albumin, although only the shape achieved statistical significance. Unlike bovine serum, cleaved albumin led to wear scars for both the POF and simulator wear tests that closely emulated the morphological features observed on explanted polyethylene tibial inserts from total knee replacements. We posit that the smaller protein fragments can more efficiently adsorb on the surfaces of both the polyethylene and the metal, thus offering protection against wear, while at the same time leading to an increase in friction, particle size, and particle elongation, as the protein fragment films interact adhesively during sliding. The results of this study have implications for pre-clinical wear testing methodology as they suggest that albumin concentration may be more pertinent than total protein concentration for wear testing polyethylene.

## 1. Introduction

It is well established that the lubricant properties play a key role in the wear of ultrahigh molecular weight polyethylene (UHMWPE or simply “polyethylene”) in prosthetic knees [1] and hips [2,3]. The total protein concentration, the albumin-to-globulin ratio, the lubricant volume turnover rate, and the protein precipitation rate, have all been found to affect the polyethylene wear rate [3]. Interestingly, bacterial contamination of the serum lubricant has been found to increase the friction and wear of polyethylene [4]. A factor that has not yet been studied, but may be of clinical relevance, is the cleavage of protein on the wear and friction of prosthetic joints. Protein cleavage is expected in the presence of an inflammatory response [5] and as a result of wear processes at the articular interface. Cleavage of proteins entails scission of the peptide chain, leading to smaller peptide fragments with exposed hydrophobic and hydrophilic moieties. It is expected to have a significant effect on the boundary lubrication of the prosthetic bearing surface by altering the molecular make-up of the boundary layer. In particular, the smaller protein fragment may lead to a less effective boundary layer and thereby higher friction and wear of the polymer.

Another motivation for the study stemmed from our observation that, in most circumstances, the bovine serum-based lubricant fails to reproduce the striated surface morphology seen on the tibiofemoral wear scar on polyethylene tibial inserts from retrieved knee implants [6–8]. Perhaps a difference of protein state in vivo versus in vitro contributes to this mismatch.

In this study, we focused on the cleavage of the bovine albumin, because this protein is the major constituent of the newborn calf serum used in wear tests to evaluate the wear of polyethylene for orthopedic applications. It accounts for approximately 42% of total protein content versus 18% for gamma globulin, according to the certificate of analysis from the manufacturer (Hyclone™, GE Healthcare Life Sciences, Logan, UT, USA).

The aim of this study was to compare the tribological behavior of polyethylene articulated against an orthopedic wrought CoCrMo alloy for three lubricants: cleaved albumin, uncleaved albumin, and newborn calf serum, which was used as a control. We hypothesized that the cleavage of albumin will significantly increase the friction and wear rate of the polyethylene sliding against an orthopedic wrought CoCrMo alloy, with a concomitant roughening of the polymer surface and the generation of larger wear debris particles.

We thus sought to answer the following research questions: (1) How does cleavage of albumin affect the friction, wear, and surface morphology of polyethylene, as tested in a pin-on-flat (POF) configuration? (2) What is the influence of protein cleavage on the size and shape distributions of the resulting polyethylene wear debris? (3) What is the effect of cleavage on the wear scar morphology of polyethylene tibial inserts tested in a knee simulator, and to what extent do they match the wear scars on retrieved polyethylene tibial inserts? In addressing these questions, we used our standard bovine serum lubricant as the reference.

## 2. Materials and Methods

### 2.1. Study Design

This study entailed two stages. In the first stage, two pin-on-flat (POF) tests were used to evaluate the effect of protein state on the friction, wear, and surface morphology of polyethylene pins articulating against CoCrMo alloy discs. Three lubricants were used: aqueous solutions of bovine serum albumin, cleaved bovine serum albumin, and bovine newborn calf serum. The first POF test entailed highly polished discs (3.1 nm Ra average), whereas the second POF used discs with a lesser finish (19.4 nm Ra average), allowing the evaluation of the effect of metal counterface roughness in conjunction with the effect of the protein state/lubricant on polyethylene wear. In the second stage, the effect of the protein state was evaluated in a knee simulator to determine if the wear morphology seen in retrieved knee insert components could be reproduced with the albumin or cleaved albumin lubricants, unlike with our standard serum lubricant.

### 2.2. Materials and Specimens

The polyethylene pins for the first pin-on-flat wear test were made of GUR 1050 UHMWPE and gamma-sterilized (2.5–3.7 Mrad) in nitrogen. The pins for the second POF test were made from non-sterilized GUR 1020 because of supply issues. The wear rate for GUR 1050 and GUR 1020 polyethylenes have been found to have similar wear rates [9] with only a minor depression of wear rate with a sterilization dose of 2.5 to 4.0 Mrad [10], and therefore were treated as equivalent. The pins had a cylindrical dual diameter geometry, with the flat contacting face having a diameter of 6.42 mm, whereas the holding end had a diameter of 9.55 mm. The average Ra surface roughness of the pins was 0.543 ± 0.014 μm (± standard deviation).

The counterface disks for the POF tests were made of low carbon wrought cobalt chrome molybdenum alloy (CoCrMo). They were 25.4 mm (1 inch) in diameter and had a thickness of 6.35 mm (0.25 inches). The active surface of all the disks were polished to a mirror finish, but the average surface roughness of the disks for the first POF test was 3.06 ± 0.17 nm Ra, versus 19.4 ± 4.4 nm Ra for the second test. The polishing was performed by the manufacturers who donated the disks (Zimmer Inc., Warsaw, IN, USA, and ESKA Implants AG, Lübeck, Germany).

The knee components used in the two knee wear tests had a posterior cruciate retaining (CR) design. In the first test, a design in current clinical use was used (NexGen Complete Knee Solution Cruciate Retaining, Zimmer, Inc., Warsaw, IN, USA). In this design, the tibial insert is machined from compression-molded GUR 1050 UHMWPE, sterilized by exposure to gamma radiation (2.5–3.7 Mrad) in nitrogen, and articulated against a CoCrMo femoral condyle. Although no longer implanted, the Miller–Galante I prosthesis (Zimmer) was used in the second test, noting that characteristic polyethylene striations have been reported for this device [6] It has a tibial insert that is made from net-shape molded Himont 1900 UHMWPE, gamma-sterilized (2.5–3.7 Mrad) in air.

The three explanted UHMWPE tibial inserts were from the Miller–Galante II prosthesis (Zimmer), a posterior cruciate retaining knee implant similar to the Miller–Galante I. They were retrieved for infection or instability from relatively young patients (Table 1). They were analyzed by optical microscopy and scanning white light interferometry (NewView 6300, Zygo Corp., Middlefield, CT, USA).

The three lubricants tested were aqueous solutions of bovine serum albumin, cleaved bovine serum albumin, and bovine newborn calf serum (Hyclone). The protein content of each lubricant was 17 g/L, which is the minimum specified in ISO Standard 14242-1:2001 for the wear testing of hip joint prostheses, and only slightly below the 19 g/L bovine serum albumin (BSA) recently recommended for synthetic synovial fluid [11]. The albumin content in the bovine serum lubricant was 7.6 g/L. The aqueous media was a buffered solution containing 9 g/L of NaCl, and 27 g/L of tris(hydroxymethyl)aminomethane adjusted to pH 7.6 with hydrochloric acid. The bovine serum albumin (96%–99%, Sigma, St. Louis, MO, USA) was cleaved with cyanogen bromide at the methionine residue, leading to cleavage positions at 111, 208, 469, and 571, with corresponding fragments of 12.6, 11.4, 29.7, 11.7, and 3.8 kD, versus 69.3 kD for the uncleaved albumin. The composition of the fragments in terms of the residue side chains character (acid, basic, hydrophilic, and hydrophobic), as well as the estimated charge on each fragment, are given in Table 2.

**Figure 1:**
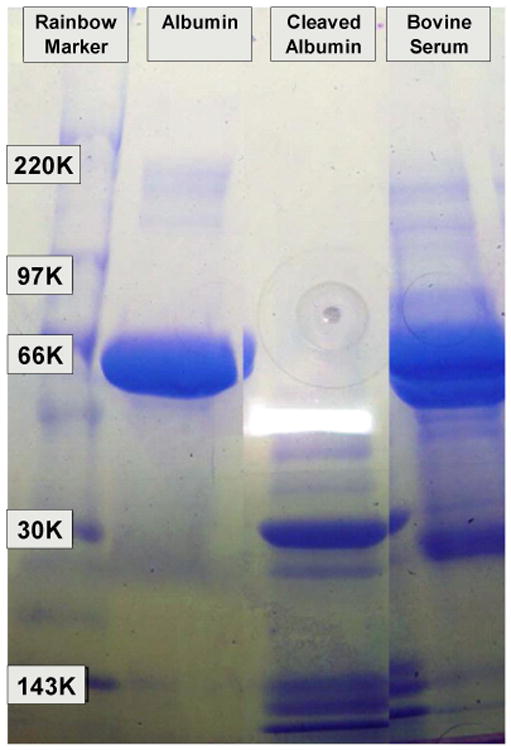
Sodium dodecyl sulfate polyacrylamide gel (SDS Page) electrophoresis of the albumin, cleaved albumin, and bovine serum. The Western blot shows that albumin has been successfully cleaved into several smaller protein fragments. Note that bovine serum carries some proteins in the size range of cleaved albumin.

The cleaved albumin was prepared as follows: Ten grams of albumin were dissolved in 50 mL of water and decolorized with 2 g activated charcoal (Sigma, C5385). The charcoal was removed by centrifugation at 10,000 g for 30 min followed by filtration through 0.1 μm filters. The albumin solution was slowly diluted with three volumes of concentrated formic acid (90%, EMD, FX0450-5, Fisher Scientific, Hampton, NH, USA). Nitrogen gas was bubbled through the solution for 5 min, then 10 g of CNBr [12] was added to the albumin–formic acid solution in a chemical hood, then the tube was flushed with nitrogen and capped. The CNBr digestion continued for 18 h at 34 °C in the chemical hood. The CNBr digest was diluted with 10 volumes of water and lyophilized twice. The dry albumin CNBr peptides were stored at −20 °C. Sodium dodecyl sulfate polyacrylamide gel (SDS Page) electrophoresis was performed on the pre-test lubricants in order to check for peptide fragment size (Figure 1).

### 2.3. Wear Tests

The pin-on-flat wear tests were conducted in a six-station pin-on-flat apparatus (OrthoPOD™; AMTI, Inc., Boston, MA, USA) in which the flat-faced UHMWPE pins slid against the polished discs; the CoCrMo counterface was used to assess the effect of the lubricant. Eight GUR 1050 UHMWPE pins (six wear, two soak controls), gamma-sterilized (2.5–3.7 Mrad), and with a face diameter of 6.4 mm, were pre-soaked in deionized distilled water prior to wear testing. During the wear test, each pin was immersed in 15 g of lubricant at 37 °C and subjected to a constant nominal contact pressure of 3 MPa along a four-sided curvilinear path, approximately 10 mm × 20 mm, or 60 mm per cycle. The path was multidirectional, with the pin surface experiencing a change in direction of 90° at each corner. On each leg of the path, the velocity was approximately constant as a result of the simultaneous arc motions of pin and disk on the OrthoPOD tribometer. The cycling frequency was 1 Hz, corresponding to a sliding speed of 60 mm/s. The test duration was one million cycles (Mc). During testing, friction was determined every 20,000 cycles for each station using six degree of freedom load cells. Every pin was subjected to 10 cycles of motion, while the tangential force was measured for 2 s at nominal contact pressure, recorded at 100 Hz and stored for later analysis on the computer. The reported friction coefficients are the average of these values for each condition (lubricant and counterface roughness) over the course of the test. Every 250,000 cycles, the pins were cleaned and weighed for gravimetric wear, and the test restarted with fresh lubricant. Two pins per lubricant were tested. When calculating the gravimetric wear, the average weight gain of the two soak control pins was used to correct for any fluid absorption by the wear pins.

The polyethylene wear surfaces were examined with a polarized light microscope at 25× magnification (DXM 1200, Nikon, Tokyo, Japan) and with a scanning electron microscope (JSM-840, JEOL USA, Inc., Peabody, MA, USA). The wear scars on three polyethylene tibial inserts retrieved from patients at post-mortem were also examined for comparison with the wear scars obtained with the POF and simulator tests.

The knee wear tests were conducted in a four-station hydraulic knee simulator (Endolab^®^ Mechanical Engineering GmbH, Rohrdorf, Germany) according to ISO14243-3, in displacement control mode to simulate level walking gait. The cycling frequency was 0.9 Hz, with a peak load of 2600 N. The lubricant was maintained at 37 °C and changed every 500,000 cycles.

### 2.4. Surface Roughness Measurements

A scanning white light interferometry microscope (NewView 6300; Zygo Corp., Middlefield, CT, USA) with a vertical resolution of up to 0.1 nm was used to determine the surface roughness of the CoCrMo discs, the polyethylene pins, and the knee tibial inserts. The measurements on the metal counterface and pins before testing were conducted using a 20× Mirau objective with a lateral resolution of 0.87 μm and a field of view of 0.702 mm by 0.527 mm; each surface was probed at 16 locations on the discs and at nine locations on the pins. The measurements on the pins after testing were performed using a 5× Michelson objective to better accommodate the relatively coarse surface features formed during wear. This objective was used with a 0.5× zoom to yield a lateral resolution of 2.9 μm and a field of view of 2.83 mm by 2.12 mm. Measurements were performed at four locations that collectively covered about 70% of the pin wear surface. Values for the following surface roughness parameters were reported: the areal Ra (arithmetic average); SRz (the average peak-to-valley areal roughness), and Rsk (roughness or skewness), a measure of the symmetry of the roughness deviations, such that a positive value of Rsk denotes the prevalence of peaks over valleys. The surface parameters were computed using MetroPro version 8.1.5 (Zygo Corp, Middlefield, CT, USA).

### 2.5. Particle Isolation and Characterization

Particle isolation from the lubricant used in the POF wear test was performed by digesting the lubricant with 10.5 M HCl for 90 min at 50 °C, followed by subsequent dilution with methanol (99.9% minimum, Optima™, Thermo Fisher Scientific, Waltham, MA, USA) and filtration through a 0.1 μm pore polycarbonate membrane (Nucleopore^®^-Track Etched, Whatman, Florham Park, NJ, USA). Photoshop CS5 (Softonic International S.A., Barcelona, Spain) and ImageJ (U.S. National Institutes of Health, Bethesda, MD, USA) were used to analyze the SEM micrographs for particle area and perimeter, from which the equivalent circle diameter (ECD) and shape ratio of the particles were calculated using the formulas in Sprecher et al. [13]. Particles were defined as rounded, elongated, or fibrillar based on their length-to-width ratio: <2, 2 to 5, or greater than 5, respectively.

### 2.6. Statistics

We evaluated the effect of lubricant and CoCrMo counterface surface roughness on friction and polyethylene wear in the POF tests using a two-way ANOVA (Design-Expert, Version 8, Stat-Ease, Inc., Minneapolis, MN, USA). Post-hoc pairwise comparisons were performed using Fisher's protected least significant difference Student t-test (Excel^®^, Microsoft Corp, Redmond, CA, USA). The effect of lubricant on the size distribution of the wear particles was analyzed using a one-way ANOVA after log-transforming the data to achieve normality. Differences in the proportion of rounded, elongated, and fibrillar particles between the three lubricants were evaluated using chi-square tests. Unless otherwise stated, the mean ± the standard deviation is given.

## 3. Results

**Figure 2:**
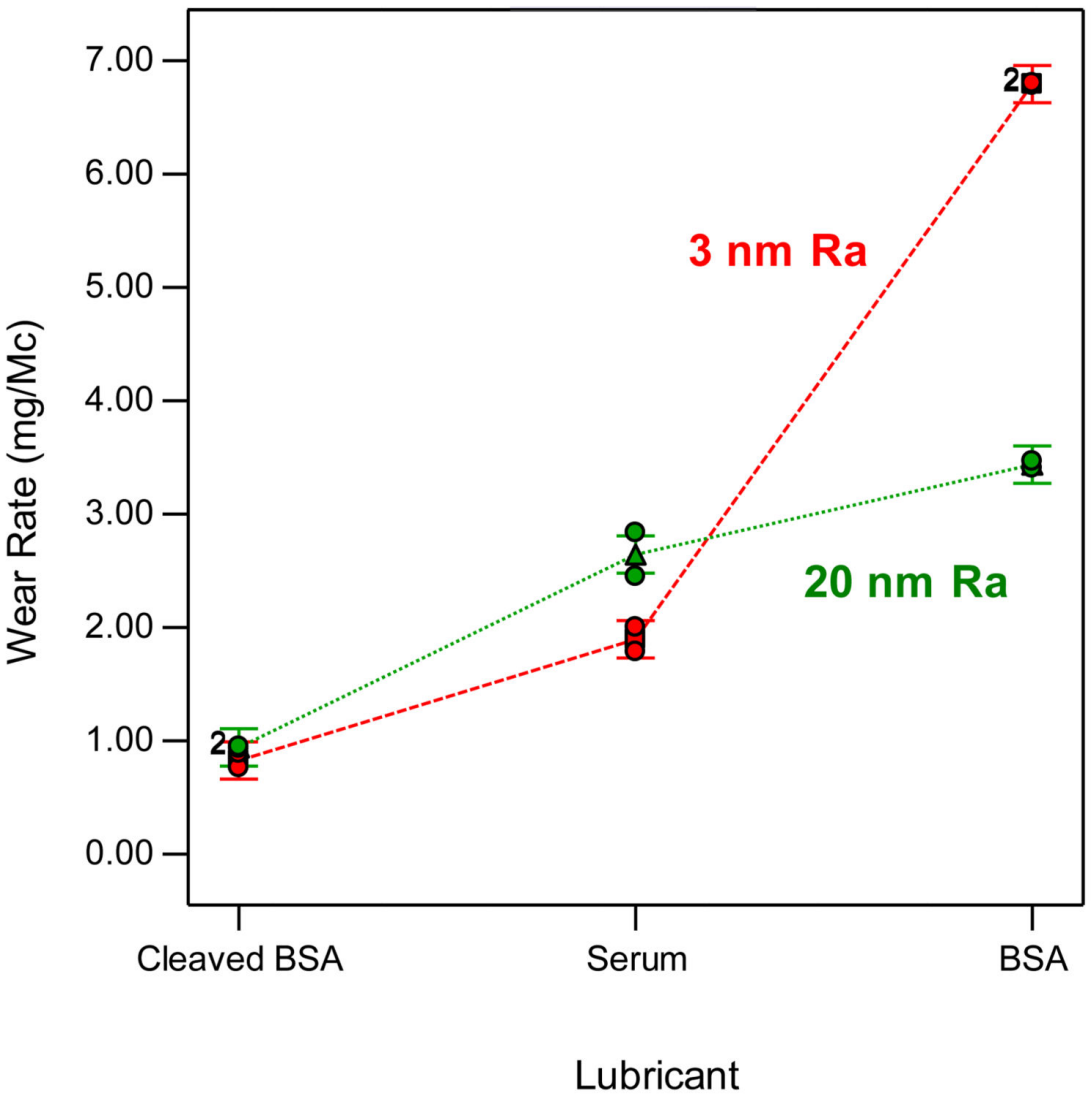
The influence of lubricant and counterface roughness on polyethylene wear as determined from POF tests. Of particular note is that for the albumin (BSA) lubricant, the higher counterface roughness (green dotted line) yielded lower wear.

For the POF test conducted against the very low roughness CoCrMo counterfaces (POF Test 1), the wear rate of UHMWPE was highest in albumin (6.79 ± 0.01 mg/Mc), lowest in the cleaved albumin (0.83 ± 0.09 mg/Mc) and intermediate in bovine serum (1.89 ± 0.15 mg/Mc) (Figure 2). These wear rates were all statistically different from each other (*p* < 0.002). Interestingly, the corresponding friction coefficients, 0.051 ± 0.002 (albumin), 0.095 ± 0.004 (cleaved albumin), and 0.064 ± 0.009 (bovine serum), scaled inversely to the wear rate; the friction coefficient for the cleaved albumin being significantly higher than for the other two lubricants (*p* < 0.012).

Against the higher roughness (19.4 nm Ra) metal disks (POF Test 2), the wear rates followed the same ranking as for the first test, with albumin producing the highest wear rate (3.44 ± 0.04 mg/Mc), followed by bovine serum (2.64 ± 0.27 mg/Mc), and cleaved albumin (0.94 ± 0.01 mg/Mc). As for POF Test 1, the cleaved albumin produced the highest friction coefficient (0.092 ± 0.006). It was significantly higher than for bovine serum (0.061 ± 0.006, *p* = 0.009), but comparable to that of albumin (0.085 ± 0.004, *p* = 0.252). The friction coefficient in bovine serum was significantly lower than in the other two lubricants (*p* < 0.018). Of particular interest is that the wear rate in the albumin lubricant against the smoother disks was decisively higher than against the rougher disks (Figure 2). Thus, both lubricant and counterface roughness had a highly significant effect (*p* < 0.0001, two-way ANOVA) on wear, and the effect of counterface roughness depended on the lubricant (*p* < 0.0001, interaction term) (Figure 2).

**Figure 3:**
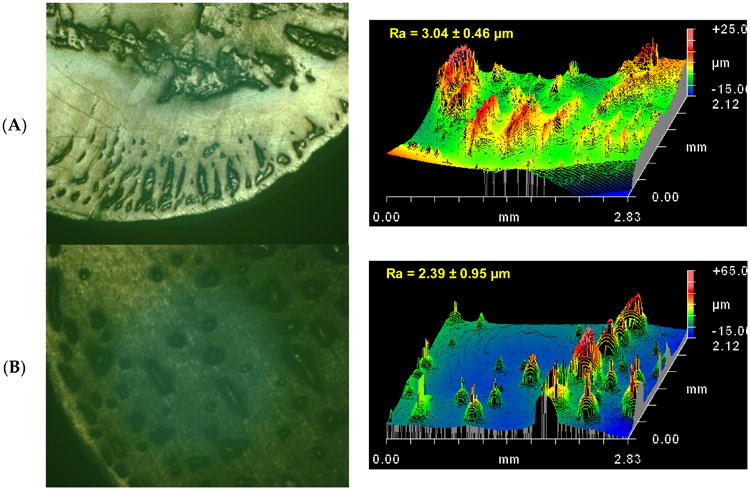

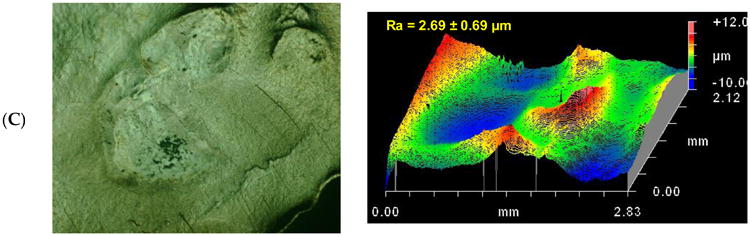
Optical micrographs and surface topography plots of the wear surfaces on the polyethylene pins wear tested against the smooth metal counterface (3 nm Ra) in (**A**) cleaved albumin; (**B**) albumin, and (**C**) bovine serum lubricants. The optical micrographs capture an area of approximately 3.6 mm × 2.9 mm. The Ra value on each topography plot is the average of all the individual measurements made on the pins ± the standard deviation. The cleaved albumin lubricant resulted in a pattern of protruding striations, whereas albumin led to numerous rounded protuberances and bovine serum yielded a largely isotropic surface of large peaks and valleys. The topography plots have different vertical scales to accommodate the characteristic features of the surfaces, even though the three surfaces have comparable Ra roughnesses.

In addition to the differences in wear and friction, the wear surface topographies also exhibited lubricant dependent differences. Whereas the pins tested in bovine serum lubricant tended to have a smooth appearance to the naked eye, the pins tested in cleaved albumin and albumin exhibited rougher looking surfaces (Figure 3). The cleaved albumin led to a dense pattern of protruding striations and rounded protuberances (3.04 ± 0.69 μm Ra), whereas albumin produced a less dense array of mostly equant protuberances (2.39 ± 0.95 μm Ra), and serum generated a largely isotropic surface with pits and protuberances (2.69 ± 0.69 μm Ra). For all three lubricants, the after-test pin surface roughness Ra was much higher than the pre-test value (0.543 ± 0.014 μm). Although the Ra values after testing were not significantly different across lubricants (*p* > 0.104), the SRz parameter was significantly lower for cleaved albumin than for albumin and serum (Table 3). The cleaved albumin lubricant also yield a significantly lower value of the skewness parameter Rsk than did the albumin and serum lubricants (Table 3). For all three lubricants, Rsk was positive, which is consistent with the observed presence of protuberances (Figure 3).

**Figure 4:**
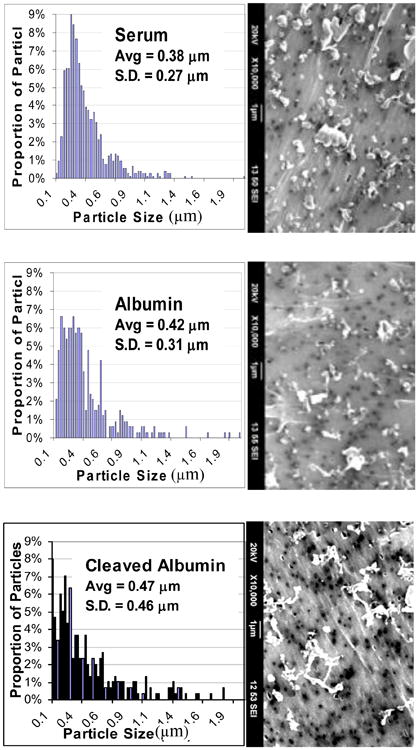
Equivalent circle diameter (ECD) size histograms and SEM micrographs of polyethylene wear debris obtained for the three lubricants in pin-on-flat (POF) Test 1.

**Figure 5:**
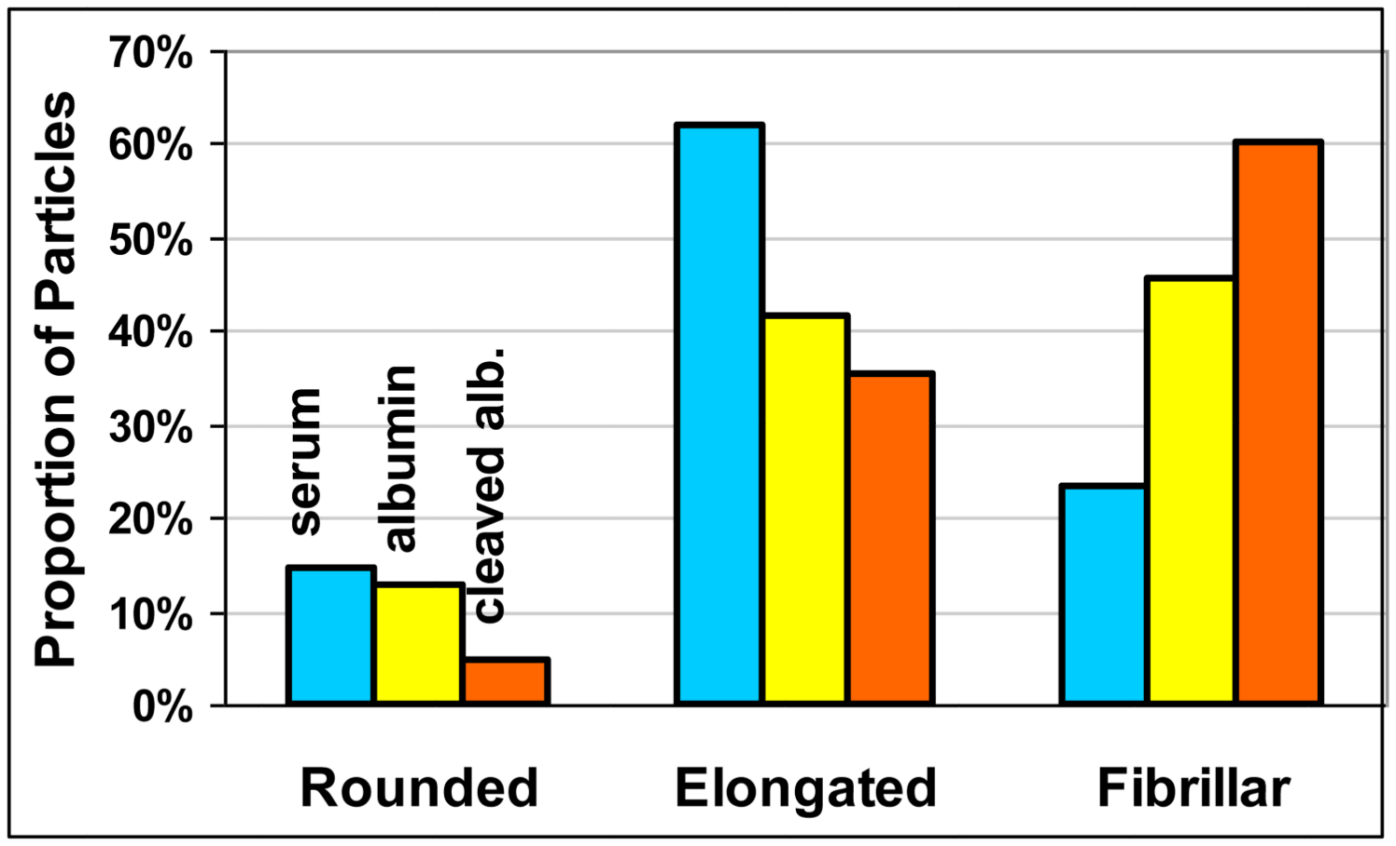
Polyethylene wear debris shape distributions obtained for the three lubricants in POF Test 1.

The morphology of the polyethylene wear debris was determined for POF Test 1, considered the base test for this study, in which the highly polished (3.1 μm Ra) CoCrMo discs were used. Of the three lubricants, serum generated the smallest average size UHMWPE particles (0.38 ± 0.27 μm), followed by albumin (0.42 ± 0.31 μm) and the cleaved albumin, which generated the largest particles (0.47 ± 0.46 μm). These differences were not statistically significant (*p* > 0.24). The particle sizes for the three lubricants followed a lognormal distribution (Figure 4), but the distribution tail toward the large sizes was more pronounced for the albumin and even more so for the cleaved albumin compared to the regular bovine serum lubricant. Thus, the proportion of particles larger than 1 μm was 3% for serum, 5% for albumin, and 11% for the cleaved albumin (*p* < 0.001). The morphology of the particles also differed markedly for the three lubricants; the serum lubricant generated the greatest proportion of rounded and elongated particles, and the proportion of fibrillar particles increased in going from serum to albumin to cleaved albumin (Figure 5). Unlike for size, these shape differences were significantly different (*p* < 0.001). The cleaved albumin lubricant generated characteristic fibrillar particles that were extremely long and had a tangled appearance.

## 4. KneeWear Tests

**Figure 6:**
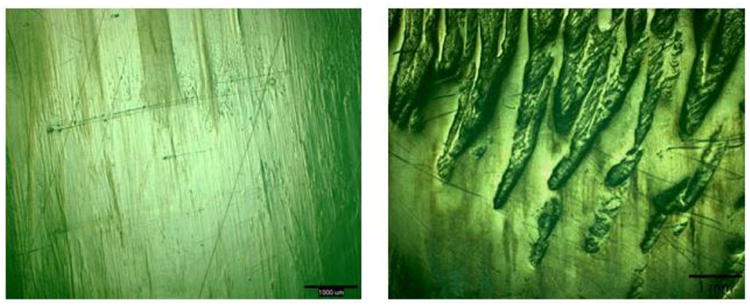
Appearance of the wear scar on a NexGen CR tibial insert after 2 Mc of testing in bovine serum (**left**), followed by 2 Mc in the albumin lubricant (**right**). The scale bars correspond to 1 mm.

In the first set of knee wear tests, performed on the NexGen CR components to determine the effect of lubricant on wear, the mean wear rate of the UHMWPE inserts increased 2.7-fold when the serum lubricant was replaced with the albumin lubricant (Table 4), following the same trend as polyethylene wear in the POD tests. There was a large concomitant 7.4-fold increase of the wear scar surface roughness, which was reflected in the surface appearance of the wear scar (Table 4). Thus, the largely polished surface obtained with the serum lubricant was replaced by a surface covered with multimillimeter long, submillimeter wide striations (Figure 6).

**Figure 7:**
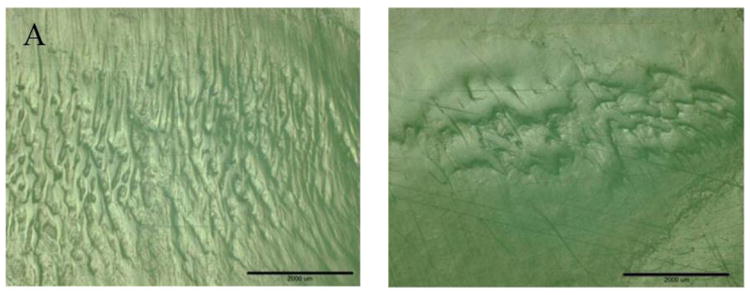

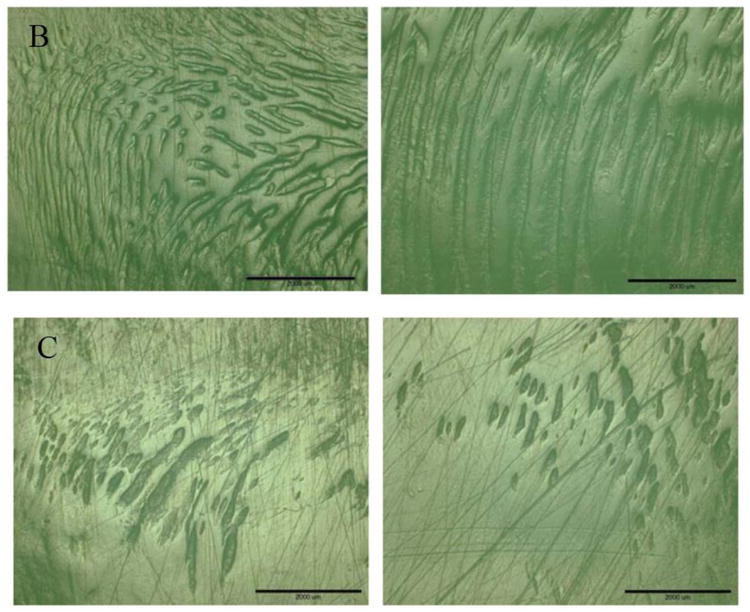
Light micrographs of wear scar areas on the medial (**left**) and lateral (**right**) articular surfaces of Miller Galante I tibial inserts tested 1.5 Mc in (**A**) cleaved albumin; (**B**) albumin; and (**C**) serum lubricants. The scale bars correspond to 2 mm.

In the second set of knee wear tests, performed on the Miller–Galante I components to determine the effect lubricant on the wear scar morphology, the albumin and cleaved albumin lubricants both yielded surfaces with generally well developed patterns of striations (Figure 7A,B), whereas the serum produced a more polished appearance, with the onset of striation (Figure 7C).

**Figure 8:**
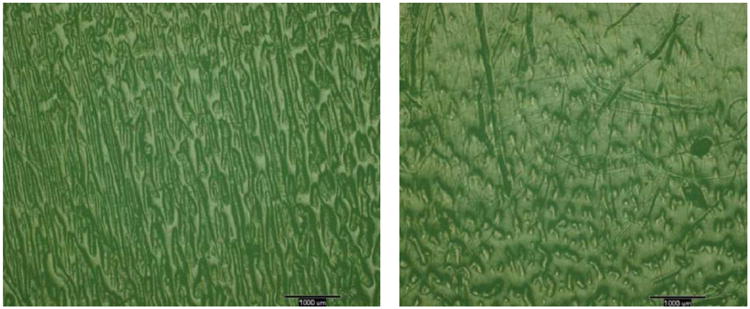
Light micrographs of wear patterns observed on the medial (left) and lateral (right) the articular surfaces of an explanted Miller–Galante II tibial insert. The scale bars correspond to 1 mm.

The wear scars of the retrieved Miller–Galante II tibial inserts exhibited pronounced striated and reticulated patterns on both the medial and lateral aspects of the insert (Figure 8). On two of the three inserts, the features appeared more elongated on the medial aspect than the lateral aspect. As seen on the inserts tested in the knee simulator, the features tended to be multimillimeter long and submillimeter wide. The mean surface roughness of these wear areas was 1 to 2 μm Ra (Table 5).

## 5. Discussion

The motivation for this study was two-fold; namely, (1) to determine the effect of protein cleavage on polyethylene wear, as such cleavage may take place clinically in the presence of an inflammatory response [5]; and (2) to determine if protein cleavage was associated with the striated surface morphology seen on the tibiofemoral wear scar on polyethylene tibial inserts from retrieved knee implants, noting that it is often absent from components tested in a bovine serum lubricant. We hypothesized that the cleavage of albumin would lead to higher friction and wear because the protein fragments would provide a less effective boundary lubrication layer than the uncleaved albumin.

Cleavage of albumin was found to have a considerable effect on all aspects of the tribological behavior of polyethylene—friction, wear, wear scar morphology, and particle morphology. The friction coefficient was indeed higher with cleaved albumin, as hypothesized, but paradoxically the wear was lower, contrary to the hypothesis. The effect was particularly marked against the ultra-smooth CoCrMo metal counterfaces (3 nm Ra), where the cleaved albumin led to an 87% increase in the friction coefficient, but a 91% decrease in the polyethylene wear rate. This inverse association of friction and wear suggests that the cleaved albumin results in the formation of boundary films that effectively shield the two wear surfaces from one another, decreasing wear, but that interact strongly, producing high friction. The greater effect found with the smooth metal counterface in terms of increasing friction but decreasing wear may arise from the more facile adsorption of the cleaved albumin fragments on the smoother surface.

Significant differences in particle size and morphology resulted from the cleavage of albumin, supporting our hypothesis. These findings, combined with the lower wear rate, higher friction coefficients, and more striated surface topography when compared to the uncleaved albumin, indicate that cleaved and uncleaved albumin lead to different wear mechanisms of polyethylene against the CoCrMo counterface. The average particle size and shape follow the albumin content of the three lubricants, indicating that the albumin protein molecule plays a key role in the wear mechanism of UHMWPE. The long fibrils and larger particles, in combination with the higher frictional forces, suggest that a non-protein mediated adhesive mechanism with polyethylene transfer may be prevalent with the cleaved albumin, whereas a micro-adhesive mechanism may prevail with albumin. The lipophilic and hydrophobic domains in albumin can presumably interact simultaneously with the polyethylene and metal asperities [14] to yield an adhesive force perhaps absent with the cleaved protein, while reducing friction by separating the two surfaces. A comparative study on the macrophage inflammatory response induced by these particles would be of clinical interest.

Of particular interest was the striking resemblance of the striated wear surface topography obtained with the cleaved albumin (Figure 7) to the topography observed on retrieved UHMWPE tibial inserts (Figure 8) [6]. Such a match was absent with the diluted bovine serum, as we have also observed in other knee wear tests using this lubricant. This result is significant because variants of this lubricant are commonly used in wear tests of hip and knee devices and are typically adjusted for the total protein content but not their individual constituents. It reveals the importance of albumin in vivo and during simulator studies.

The stark difference in wear and friction between albumin and cleaved albumin can be explained by the difference in the adsorption mechanism of the two protein states, based on the extension of an adsorption model by Widmer et al. [14]. Albumin affinity for the hydrophilic metal surface exposes the hydrophobic groups that can bond with the hydrophobic UHMWPE surface. This results in the attachment of the albumin molecule to both metal and UHMWPE surfaces. This interaction results in reduced friction by separating the surfaces, but also to increased wear due to effective removal of UHMWPE where polymer–protein–metal adhesion has occurred. This adhesive wear effect was more pronounced against the smoother metal disk, which fits the proposed model. Thus, when a significant fraction of the valleys on the metal counterface have depths that exceed the size of the albumin molecules, the proportion of albumin bridging molecules is reduced, leading to reduced wear. Given the reported size of 14 nm × 4 nm × 4 nm for the albumin molecule [15], this effect is expected when the counterface roughness has an Ra significantly greater than 14 nm, as observed here (Figure 2). For the cleaved BSA, on the other hand, the protective effect of the relatively dense protein film is independent of counterface roughness, and therefore so is wear (Figure 2), at least within the limits of roughness tested here (3 and 20 nm).

In contrast to the albumin, the cleaved albumin consists of numerous relatively short peptide chains with both hydrophilic and hydrophobic residue sequences that can adsorb to either the polymer or metal surface separately, and are thus less likely to form a molecular bridge that can simultaneously attach to the polymer and metal surfaces and lead to wear. At the same time, the smaller size of the cleavage fragments leads to greater coverage density than the much larger albumin molecules which sterically hinder each other on the surface. The greater coverage with the cleavage fragments in turn produces higher frictional forces as these denser protein films interact through hydrogen bonding and van der Waals forces. This coverage, however, reduces wear by preventing direct metal–polyethylene asperity contact. It should be noted that four of the five cleaved albumin residues have very similar proportions of acidic, hydrophilic and hydrophobic residues (Table 2), and therefore cannot, on that basis, be assigned preference for metal or polymer. The fifth fragment has a higher proportion of hydrophobic residues (61%) than the other four fragments (45%–46%), which may favor adsorption to polyethylene, but it also carries a negative charge, which may favor adsorption to the metal surface. It may therefore readily adsorb to both surfaces.

This study has several limitations. First, GUR 1050 UHMWPE was used for the first POF test, whereas GUR 1020 UHMWPE was used for the second test. However, these two materials have been shown to have similar wear rates [9,10], and therefore were treated as practically equivalent. In addition, in a separate POF experiment with non-sterilized GUR 1020 as the polyethylene and albumin as the lubricant against a similarly smooth metal disk (Ra = 4.1 ± 1.2 nm) as in this study, we found a wear rate of 8.0 ± 2.3 mg/Mc for the polyethylene that is comparable to that GUR 1050 in POF 1 [16]. More importantly, the roughness of the disks differed between POF Tests 1 and 2. This difference was captured, in that roughness was included as a factor in the model. Second, this study focused on the effect of albumin on wear and friction and ignored the effect of other proteins (e.g., gamma-globulins) on wear, friction, and scar morphology. Hyaluronic acid and phospholipids present in synovial and periprosthetic fluids [17], components that can influence friction and wear [11,18], were also not taken into account. Third, only retrieved tibial inserts from the Miller–Galante II prosthesis, a historical design, were examined for this study. However, striations have been reported on multiple designs by us and others [6,8,19,20], and an unpublished database review in 2003 involving two institutions (Rush University Medical Center, Chicago, IL, and Good Samaritan Medical Center, West Palm Beach, FL, USA) found the striated pattern on 65% of 162 tibial inserts from five manufacturers, made from conventional polyethylene. More recently, during an analysis of 81 retrieved NexGen CR components, which were machined from compression molded GUR 1050, striations were found on the articular surface of 61 components (i.e., >75%) [21].

## 6. Conclusions

This study demonstrated that the cleavage of albumin has a pronounced effect on the wear, friction, and wear scar morphology of polyethylene sliding against a cobalt chrome alloy counterface. It led to a decrease in wear with a paradoxical increase in friction. Cleavage of albumin also led to a more filamentous polyethylene wear debris. Of particular significance, the cleavage of albumin led to wear scars that closely emulated the morphological features observed on explanted polyethylene tibial inserts from total knee replacements. We posit that the smaller protein fragments can more efficiently adsorb on the surfaces of both the polyethylene and the metal, thus offering protection against wear, while at the same leading to an increase in friction as the fragments interact adhesively during sliding. The uncleaved albumin may also increase wear through an adhesive bridging mechanism across the interface where it bonds to both polymer and metal, thus efficiently transmitting a tearing shear force against the polymer. This study suggests that the albumin concentration may be as relevant as the total protein concentration when using a bovine serum lubricant in wear tests of polyethylene implants. A standardized lubricant made from known base ingredients and in which the proteins are protected from biological degradation could lead to more reproducible and consistent wear results [4]. In a future study, it would be of interest to investigate the effect of lowered pH that can occur during inflammation and infection [22–25], jointly with protein cleavage.

## Figures and Tables

**Table 1:** Demographic information for the retrieved tibial inserts.

Patient No.	Side	Time (Months)	Age (Years)	Gender	Reason for Removal
1	R	27	66	M	Infection
2	L	60	59	F	Infection
3	L	13	55	F	Instability

**Table 2:** Residue type make-up of the cleaved albumin fragments and the estimated charge on each fragment.

Residue Type	Fragment				

	1	2	3	4	5
A−	18%	20%	16%	15%	17%
B+	10%	14%	16%	15%	8%
PHI	25%	21%	23%	25%	14%
PHO	46%	45%	45%	45%	61%
Number of Residues	87	97	261	102	36
Charge	negative	negative	negative	neutral	negative

A− = acidic, B+ = basic, PHI = hydrophilic, PHO = hydrophobic.

**Table 3:** Surface roughness parameter values and their comparison across the three lubricants for the wear surfaces of the polyethylene pins tested against the smooth (3 nm) CoCrMo alloy counterface. Mean values are given with the standard deviation in parentheses. *p* values ≤ 0.05 are in bold.

Lubricant	Ra (μm)	SRz (μm)	Rsk
Cleaved Albumin	3.04 (0.69)	36.6 (31.4)	1.34 (2.50)
Albumin	2.39 (0.95)	73.4 (12.5)	5.56 (2.49)
Serum	2.69.(0.69)	63.1 (31.4)	2.65 (2.50)

***p* values for the Pairwise Comparisons of the Three Lubricants** *			

Cleaved Albumin vs. Albumin	0.104	0.000	0.000
Cleaved Albumin vs. Serum	0.258	0.041	0.159
Albumin vs. Serum	0.477	0.403	0.035

* Obtained using a two-tailed *t*-test applied to the individual measurements made on the pins.

**Table 4:** Wear rates, wear scar surface roughness and appearance for tibial inserts articulated against CoCrMo femoral components in bovine serum and albumin lubricants.

Parameter	Bovine Serum	Albumin Lubricant	Ratio
Wear Rate (mg/MC)	1.47	4.01	2.7
Wear Scar Ra (μm)	0.168 ± 0.1	1.25 ± 0.68	7.4
Wear Appearance	Mostly polished	Striations and protuberances	-

**Table 5:** Mean surface roughness Ra of the wear scar of the three retrieved Miller–Galante II tibial inserts.

Patient No.	Ra (μm)
1	1.24 ± 0.45
2	2.08 ± 1
3	1.05 ± 0.3
New Insert	0.13

## References

[1] Schwenke T, Kaddick C, Schneider E, Wimmer MA (2005). International Standards Organization Fluid composition impacts standardized testing protocols in ultrahigh molecular weight polyethylene knee wear testing. Proc Inst Mech Eng H.

[2] Liao YS, Benya PD, McKellop HA (1999). Effect of protein lubrication on the wear properties of materials for prosthetic joints. J Biomed Mater Res.

[3] Wang A, Essner A, Schmidig G (2004). The effects of lubricant composition on in vitro wear testing of polymeric acetabular components. J Biomed Mater Res B.

[4] Wimmer MA, Sah R, Laurent MP, Virdi AS (2013). The effect of bacterial contamination on friction and wear in metal/polyethylene bearings for total joint repair—A case report. Wear.

[5] Rengel Y, Ospelt C, Gay S (2007). Proteinases in the joint: Clinical relevance of proteinases in joint destruction. Arthritis Res Ther.

[6] Wimmer MA, Andriacchi TP, Natarajan RN, Loos J, Karlhuber M, Petermann J, Schneider E, Rosenberg AG (1998). A striated pattern of wear in ultrahigh-molecular-weight polyethylene components of Miller-Galante total knee arthroplasty. J Arthroplast.

[7] Wimmer MA (1999). Wear of the Polyethylene Component Created by Rolling Motion of the Artificial Knee Joint.

[8] Knowlton CB, Wimmer MA (2016). Relationship of Surface Damage Appearance and Volumetric Wear in Retrieved TKR Polyethylene Liners. J Biomed Mater Res B.

[9] Hunt BJ, Joyce TJ (2016). A Tribological Assessment of Ultra High Molecular Weight Polyethylene Types GUR 1020 and GUR 1050 for Orthopedic Applications. Lubricants.

[10] Tipper JL, Galvin AL, Ingham E, Fisher J Comparison of the Wear, Wear Debris and Functional Biological Activity of Non-crosslinked and Crosslinked GUR 1020 and GUR 1050 Polyethylenes used in Total Hip Prostheses.

[11] Bortel EL, Charbonnier B, Heuberger R (2015). Development of a Synthetic Synovial Fluid for Tribological Testing. Lubricants.

[12] Kopoldová J, Liebster J, Gross E (1967). Radiation chemical reactions in aqueous solutions of methionine and its peptides. Radiat Res.

[13] Sprecher CM, Schneider E, Wimmer MA (2004). Generalized Size and Shape Description of UHMWPE Wear Debris—A Comparison of Cross-Linked, Enhanced Fused, and Standard Polyethylene Particles. J ASTM Int.

[14] Widmer MR, Heuberger M, Vörös J, Spencer ND (2001). Influence of polymer surface chemistry on frictional properties under protein-lubrication conditions: Implications for hip-implant design. Tribol Lett.

[15] Wright AK, Thompson MR (1975). Hydrodynamic structure of bovine serum albumin determined by transient electric birefringence. Biophys J.

[16] Dwidvedi Y (2009). Effect of Proteins on the Wear of UHMWPE (Ultra High Molecular Weight Polyethylene). Master's Thesis, University of Illinois at Chicago, Chicago, IL, USA.

[17] Fam H, Bryant JT, Kontopoulou M (2007). Rheological properties of synovial fluids. Biorheology.

[18] Park JB, Duong CT, Chang HG, Sharma AR, Thompson MS, Park S, Kwak BC, Kim TY, Lee SS, Park S (2014). Role of hyaluronic acid and phospholipid in the lubrication of a cobalt-chromium head for total hip arthroplasty. Biointerphases.

[19] Harman MK, Banks SA, Hodge WA (2001). Polyethylene damage and knee kinematics after total knee arthroplasty. Clin Orthop Relat Res.

[20] Perinchief RS, Muratoglu OK, Spiegelberg SH, Harris WH (2001). On the surface morphology of surgically retrieved UHMWPE tibial knee inserts. Trans Soc Biomater.

[21] Rad EM (2015). Volumetric Wear Assessment and Characterization of Striated Pattern of Retrieved UHMWPE Tibial Inserts. Master's Thesis.

[22] Jebens EH, Monk-Jones ME (1959). On the Viscosity and pH of Synovial Fluid and the pH of Blood. J Bone Jt Surg Br.

[23] Cummings NA, Nordby GL (1966). Measurement of synovial fluid pH in normal and arthritic knees. Arthritis Rheum.

[24] Treuhaft PS, McCarty DJ (1971). Synovial fluid pH, lactate, oxygen and carbon dioxide partial pressure in various joint diseases. Arthritis Rheum.

[25] Stafford G, Akmal M, Mitchell-Hynd C, Skinner J, Bentley G Synovial Fluid pH as an Indicator of Infected Joint Arthroplasty. http://www.orthoteers.org/(S(4rn2454fldm1014g5dk4wh2s))/owls.aspx?section=37&article=196.

